# Population structure of *Venturia inaequalis*, a causal agent of apple scab, in response to heterogeneous apple tree cultivation

**DOI:** 10.1186/s12862-018-1122-4

**Published:** 2018-01-19

**Authors:** Monika Michalecka, Sylwester Masny, Thibault Leroy, Joanna Puławska

**Affiliations:** 1 Department of Phytopathology, Research Institute of Horticulture, Konstytucji 3 Maja 1/3, 96-100 Skierniewice, Poland; 2 UMR 1202 BIOGECO, INRA, F-33610 Cestas, France

**Keywords:** Apple cultivar resistance, Disease emergence, Secondary contact, Sympatric populations

## Abstract

**Background:**

Tracking newly emergent virulent populations in agroecosystems provides an opportunity to increase our understanding of the co-evolution dynamics of pathogens and their hosts. On the one hand host plants exert selective pressure on pathogen populations, thus dividing them into subpopulations of different virulence, while on the other hand they create an opportunity for secondary contact between the two divergent populations on one tree. The main objectives of the study were to explore whether the previously reported structure between two *Venturia inaequalis* population types, virulent or avirulent towards *Malus* x *domestica* cultivars carrying *Rvi6* gene, is maintained or broken several years after the first emergence of new virulent strains in Poland, and to investigate the relationship between ‘new’ and ‘native’ populations derived from the same commercial orchards. For this purpose, we investigated the genetic structure of populations of the apple scab fungus, occurring on apple tree cultivars containing *Rvi6*, *Rvi1* or *Rvi17* resistance gene or no resistance at all, based on microsatellite data obtained from 606 strains sampled in 10 orchards composed of various host cultivars.

**Results:**

Application of genetic distance inferring and clustering methods allowed us to observe clear genetic distinctness of the populations virulent towards cultivars carrying *Rvi6* gene from the *Rvi6-*avirulent populations and substructures within the *Rvi6-*group as a consequence of independent immigration events followed by rare, long-distance dispersals. We did not observe such a structuring effect of other genes determining apple scab resistance on any other populations, which in turn were genetically homogenous. However, in two orchards the co-occurrence of strains of different virulence pattern on the same trees was detected, blurring the genetic boundaries between populations.

**Conclusions:**

Among several resistance genes studied, only *Rvi6* exerted selective pressure on pathogens populations: those virulent toward *Rvi6* hosts show unique and clear genetic and virulence pattern. For the first time in commercial *Malus* x *domestica* orchards, we reported secondary contacts between populations virulent and avirulent toward *Rvi6* hosts. These two populations, first diverged in allopatry, second came into contact and subsequently began interbreeding, in such way that they show unambiguous footprints of gene flow today.

**Electronic supplementary material:**

The online version of this article (10.1186/s12862-018-1122-4) contains supplementary material, which is available to authorized users.

## Background

Fungal pathogens may affect plant physiology and reduce plant fitness by reducing the quality and quantity of seeds or fruits. Consequently, diseases caused by fungal pathogens are a vital component of global yield losses and therefore require intensive crop protection practices against pathogens, including intensive chemical use. However, the increasing demand for organic and chemical residue-free food requires the development of alternative protection practices, such as genetic pest control via plant breeding. This is even more important for agrosystems in which crop rotation is impossible, such as the perennial apple, grape or citrus tree crops. *Venturia inaequalis* (Cooke) G. Winter is a haploid fungus from the *Ascomycotina* class that is responsible for the most damaging apple disease reported in almost all apple-growing regions - apple scab. Its annual cycle includes sexual reproduction on infected apple leaf litters in the winter followed by several cycles of asexual reproduction during the apple growing season, what makes disease management challenging. Disease control predominantly relies on leaf litter management and repeated fungicide applications in the spring [1, 2].

Most genetic pest controls for *V. inaequalis* rely on gene-for-gene interactions [3], such that the infection outcome is determined by the interaction between the products from a specific locus in the plant (the main resistance *R* gene) and a gene from the pathogen (avirulence gene). In this kind of interaction, the product of a given avirulence gene is recognized by the plant harbouring the matching resistance allele of the corresponding *R* gene. If a plant harbours the susceptibility allele or if the pathogen has the alternative virulence allele, infection occurs. To date, 17 main *R* genes that determine resistance against apple scab have been identified within the *Malus* species [4]. For example, in Poland, the first scab resistant cultivars were imported from the USA in the early 1970s. The *R* gene *Rvi6* (previously named *Vf*, [4], originating from *M. floribunda*) has been widely introgressed in cultivars of *M.* x *domestica* since that time, while the *Rvi1* (previously named *Vg*, [4], originating from ‘Golden Delicious’ cultivar of *M.* x *domestica*) and *Rvi17* (previously named *Va1*, [4], originating from ‘Antonovka’ cultivar of *M.* x *domestica*) cultivars have been registered in Poland in the 1990s. On the other hand, a number of apple resistance genes that were recently introgressed into *M. x domestica* cultivars from wild genetic resources of *Malus* were overcome by the *V. inaequalis* fungus, as reported from many areas of apple cultivation in Europe ([5–7] and others). However, in Poland, the *Rvi6* cultivars (mainly Topaz, Rubinola and Freedom) were considered as free from *V. inaequalis* infections until the first report of apple scab symptoms in the Topaz cultivar in 2009 [8]. In the subsequent years, apple scab was observed on other *Rvi6* cultivars, but the severity of the disease varied.

Previous population genetic studies have shown that apple resistance genes might induce specialization in pathogen populations, resulting in host-related adaptations [9, 10]. The presence or absence of a single resistance gene in the host plant (*Rvi6*) reportedly has a very strong effect on the structure of populations that infect both genotypes that can be generally maintained, even at the orchard scale [11, 12]. Recent demographic history studies [13] have revealed that the *Rvi6* and non-*Rvi6* *V. inaequalis* populations diverged several thousand years ago (8000–50,000 years) with no detectable gene flow between the two lineages. Based on extensive studies comprising populations at the European scale, Lemaire et al. found strong statistical support for a scenario in which the populations infecting the current *Rvi6* varieties present in the agroecosystem emerged from existing populations present on the wild progenitor Japanese crabapple *M. floribunda*, which was further followed by several migration events of *Rvi6-*virulent individuals to European orchards. In a French orchard composed of both *Rvi6* and non-*Rvi6* hosts, Leroy et al. demonstrated that secondary gene flow between both populations is possible in nature, leading to both genetic and epidemiological changes [14].

Based on observations of disease symptoms in *Rvi6* cultivars, we concluded that this mechanism of resistance has also been overcome in Poland. Since little is known about the genetic structure of the newly observed pathogen populations affecting *Rvi6* cultivars in Poland or their relationship with ‘native’ *V. inaequalis* populations, we wanted to assess the occurrence and characterize these pathogens in several organic orchards. One of our main goals was to assess the maintenance of reproductive isolation between populations in different commercial organic orchards, including those in which *Rvi6* and non-*Rvi6* cultivars occur sympatrically. This assessment would elucidate the evolutionary potential of fungal populations which diverged in allopatry for some period of time, what should be reflected in their genetic distinctness, yet now are observed in the same orchards, experiencing the secondary contacts. Moreover, we wanted to explore whether monogenic resistances other than the *Rvi6* present in *Malus x domestica* cultivars in Poland could differentiate pathogen populations. We wanted to explore the genetic relationship between the *Rvi6-*virulent populations and other virulent pathogen populations, particularly those affecting apple cultivars containing *Rvi17* (*Va1*), *Rvi1* (*Vg*) or those without any known *R* genes by tracking the recombination possibilities between them. To reach this goal, we analysed 633 infected leaves, collected in 10 orchards across Poland and genotyped these samples at 11 microsatellite loci, since microsatellite markers were found as a good method used for *V. inaequalis* genotyping, suitable for inference about differentiation at fungal population level [2, 10, 11, 15, 16].

## Methods

### Fungal material and DNA isolation

**Fig. 1:**
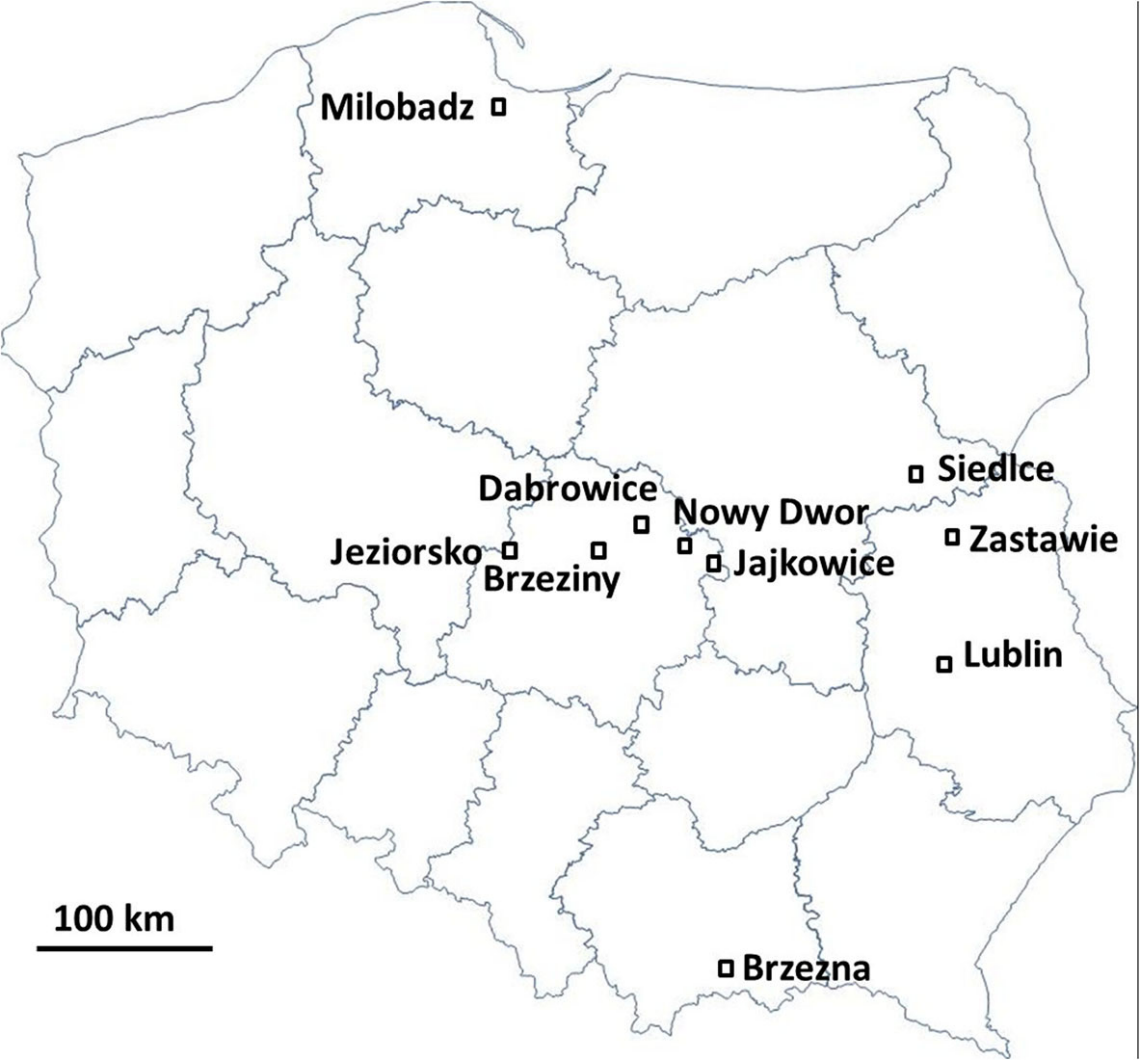
Schematic map of Poland showing the location of the orchards in which the *V. inaequalis* populations were sampled

During the late spring and early summer of 2012–2014, two commercial chemically controlled, six organic fungicide-free and two with mixed type of control apple tree orchards located in the central, southern and northern parts of Poland (Fig. 1, Table 1) were sampled, including the orchard in which the first *Rvi6*-virulent strains were reported in Poland (Lublin). In all the orchard trees of *Malus* x *domestica* were sampled, and one population was collected from crab apple trees - F1 seedling of *Malus* x *zumi* var. ‘Colocarpa’ originated from open pollination (OZD, Dabrowice). Infected leaves with clear scab lesions were randomly collected from mono-R-genic cultivars (*Rvi1*, *Rvi6* or *Rvi17*) and from cultivars without any known sources of apple scab resistance (hereafter *Rvi0*). Once the leaves were air dried, single and distinct scab lesions were cut from the leaves, placed into Eppendorf tubes and frozen at − 20 °*C. prior* to DNA extraction, the samples were submerged in liquid nitrogen and homogenized using a sterile mortar and pestle. The total DNA was extracted using the GeneMatrix Plant & Fungi DNA Purification Kit (EURx, Gdańsk, Poland) according to the manufacturer’s instructions and eluted with 100 μl of the provided elution reagent. In total, DNA from 20 orchard populations, each comprising of 30–35 single scab lesions, were subjected to further analyses.

**Table 1:** The names and origins of the *V. inaequalis* populations studied and a summary of the significant results comprising the genetic polymorphism values

*R* gene	Orchard location, cultivar	Control type	Population name	n	na	cf	A	AR	AP	*H*_*D*_
0^1^	Dabrowice, F1 seedling of *Malus* x *zumi* var. ‘Colocarpa’	organic	OZD	32	32	0.0	6.73	6.25	1	0.690
0	Lublin, Gala	chemical	MGL	31	31	0.0	6.64	6.19	2	0.688
0	Lublin, Paulared	chemical	PAL	32	31	3.1	7.09	6.66	6	0.740
0	Nowy Dwor, Ligolina	organic	LND	32	32	0.0	6.82	6.38	4	0.674
0	Nowy Dwor, Delbard Jubile	organic	DND	30	30	0.0	6.73	6.36	3	0.699
*Rvi1*	Brzezna, Golden Delicious	chemical	GDB	32	32	0.0	6.45	5.99	2	0.662
*Rvi1*	Jajkowice, Golden Delicious	chemical	GDJ	32	32	0.0	6.91	6.37	0	0.699
*Rvi1*	Milobadz, Golden Delicious	chemical	GDM	32	32	0.0	7.00	6.47	5	0.672
*Rvi6*	Nowy Dwor, Enterprise	organic	END	32	25	21.9	3.73	3.63	2	0.566
*Rvi6*	Nowy Dwor, Rajka	organic	RND	31	26	16.1	4.45	4.32	1	0.587
*Rvi6*	Brzeziny, Rubinola	organic	RUB	32	30	6.3	3.36	3.22	0	0.503
*Rvi6*	Brzeziny, Topaz	organic	TOB	32	32	0.0	3.55	3.34	0	0.508
*Rvi6*	Jeziorsko, Topaz	organic	TOJ	31	26	16.1	3.64	3.55	1	0.460
*Rvi6*	Jeziorsko, Biogolden	organic	BGJ	32	31	3.1	3.64	3.44	1	0.413
*Rvi6*	Lublin, Ariwa	organic	ARL	32	32	0.0	4.09	3.93	0	0.535
*Rvi6*	Lublin, Gold Milenium	organic	GML	31	28	9.7	3.18	3.06	0	0.431
*Rvi17*	Brzezna, Antonovka	organic	ABR	32	32	0.0	7.09	6.40	7	0.620
*Rvi17*	Siedlce, Antonovka	organic	ASI	32	29	9.4	8.09	7.68	7	0.762
*Rvi17*	Zastawie, Antonovka	organic	AZL	32	32	0.0	6.18	5.80	3	0.655
*Rvi17*	Dabrowice, Reglindis	organic	RED	31	31	0.0	6.64	6.22	1	0.699

*n* number of individuals genotyped

*na* number of individuals analysed, after clone-correction

cf clonal fraction in %

*A* mean number of alleles per locus

*AR* allelic richness

*AP* number of private alleles

*H*_*D*_ unbiased gene diversity, estimated following Nei 1987

0^1^ cultivar without known *R* gene, referred in the text as ‘*Rvi0*’

### Amplification of *V. inaequalis* microsatellite markers

The DNA from each sample was genotyped at 11 microsatellite loci. The amplifications were performed in one simplex and five multiplexes PCRs. Each forward primer in the applied primer sets (1tc1a, 1tc1b, 1aac3b, 1tc1g [17], Vitcca7/P, Vica9/152, Vitg11/70, Viga7/116, Vica9/X, Vicacg8/42 [15] and M42 [11]) was labelled with a non-radioactive fluorescent dye (HEX, TAMRA or FAM, Genomed SA, Warsaw, Poland). The reactions were performed in a total volume of 15 μl containing 1–10 ng of DNA, 0.2 mM of each dNTP, 333.3 nM of each primer, 0.6 U of GoTaq® G2 Flexi DNA Polymerase (Promega Corp., Madison, USA), 1 x optimized reaction buffer (Promega), 1.5 mM MgCl_2_ and double distilled water. The conditions for each reaction consisted of an initial denaturation at 95 °C for 3 min, followed by 40 cycles of denaturation at 95 °C for 30 s, annealing at 58 °C for 30 s (50 °C for the 1tc1g primer set), polymerisation at 72 °C for 30 s and a final extension step at 72 °C for 5 min. The amplicons sizes were scored using an ABI PRISM 310 Genetic Analyzer with GeneScan™ 500 ROX™ (Applied Biosystems, Foster City, California, USA) as an internal size standard and analysed using Peak Scanner Software 1.0 (Thermo Fisher Scientific). Strains with profiles showing more than one clear signal per microsatellite marker were excluded, in order to avoid the possibility of genotyping of multiple infections in the same area of a leaf (i.e. several genotypes in a single observable scab lesion).

### Dataset preparation

Because the samples were collected during the asexual phase of the *V. inaequalis* life cycle, repeated identical multilocus genotypes were identified using the GenoType application [18] and discarded from the data set. A clone corrected dataset was used for the analyses.

### Clustering methods

To determine the most likely number of populations represented by the samples and to assess the levels of differentiation, five clustering and assignment methods were used.

1. A chord distance matrix between the populations [19] was created from the dataset using MSA programme [20] that presented the geometric distance between the data without biological assumptions. Based on the matrix, principal coordinate analysis (PCoA) was performed using GenAlEx 6.5 programme [21] for the whole data set and for the *Rvi6* and non-*Rvi6* populations separately.
2. Individuals were assigned to regional sample groups using GeneClass 2.0 [22]. The probability of individuals coming from a group other than the one in which they were primarily assigned (i.e. *Rvi6*-group, *Rvi1*-group, *Rvi17*-group or *Rvi0*-group) was calculated using the standard Bayesian criteria described by Rannala and Mountain [23] and by simulating 1000 individuals per population using the method provided by Paetkau et al. [24]. Individuals were assigned to the population group that had the highest probability of being their source.
3. The STRUCTURE 2.3.4 programme [25], which implements the Bayesian model-based clustering method for inferring population structures, was used to assign individuals to a specific number of clusters (K). The implemented models assumed that the loci within populations are at Hardy-Weinberg equilibrium and linkage equilibrium. To estimate the optimal number of clusters, K values ranging from 1 to 15, with ten repetitions for each value, were tested to examine the likelihood convergence values for each K value. After the initial burn-in period of 5 × 10^4^ iterations, 1 × 10^4^ Monte Carlo Markov Chain schemes were performed for each run. No prior information regarding individual samples related to their location or their host was assumed, and a model with correlated allele frequencies and admixture among the populations was applied. The number of populations that best represented the observed data under the model implemented was determined by maximizing the estimated ln-likelihood of the results obtained for the different K values and the ΔK index, which is based on the rate of change in the ln-likelihood of the data between successive K values, to find the best K value estimates [26] using the online programme STRUCTURE HARVESTER. The ΔK values were plotted against K values ranging from 2 to 15, and the optimal cluster solution indicated by the programme represents the K value for which the highest peak was observed. The proportion of ancestry represented by the Q-value matrices obtained for K values ranging from 2 to 5 were summarized using the CLUMPAK package [27] to obtain the average individual membership coefficients in the inferred clusters.
4. Another programme TESS 2.3.1, implementing an individually based spatially explicit Bayesian clustering algorithm for population genetic studies [28, 29], was used to determine the admixture proportion for the individuals. In the analysis, individual assignments to the different genetic clusters (K values from 2 to 10) were simulated 100 times using an admixture model [30] with 5000 Markov Chain Monte Carlo (MCMC) sweeps and 1000 burn-in MCMC sweeps. The deviance information criterion (DIC), a statistical measure of the model prediction capabilities, was computed by TESS for each simulation. A comparison of the best simulations based on the DIC values per K value was used to determine the most likely number of genetic clusters for each analysis. Mean DIC values, obtained for the ten runs with the lowest DIC values, were plotted against K values ranging from 2 to 10. The optimal cluster solution indicated by the programme represents the K value for which the inflection point was observed. The proportion of ancestry represented by the Q-values matrices of the top 10% simulations per K value (i.e., the lowest DIC value per K value) were summarized using the CLUMPAK package.
  Based on the CLUMPAK outputs obtained from TESS and STRUCTURE Q-values, graphical presentations of the average individual membership coefficients were performed using R script available with TESS software [31]. The estimated membership probabilities for each individual are presented in a bar chart, allowing for the inference of whether individuals shared ancestry with multiple clusters and to verify the fraction of individual genotypes that were assigned to each cluster according to the implemented admixture model.
5. For the purpose of comparison with Bayesian-based clustering algorithms, the clustering method based on distance matrix was applied. GDA program [32] was used to obtain matrices of pairwise estimates of genetic distance among 20 populations (according to Nei, 1978, [33]). Based on the obtained matrices and using Neighbour-joining (NJ) and UPGMA algorithms, the program constructed dendrograms, which were subsequently visualized using TreeViewX free software.

### Standard genetic diversity analyses

For each population, the mean number of alleles per locus and the number of private alleles (i.e., alleles that were found only in a single population among a broader collection of populations) were estimated using the GDA, allelic richness (calculated as the average number of alleles per locus) and unbiased average gene diversity (*H*_*D*_, [34]) using FSTAT 2.9.3.2 [35], and allele frequency using ARLEQUIN 3.5.1.2 [36] programmes.

### Analysis of genetic variation between populations and groups of populations

Population genetic structure was examined by means of a hierarchical analysis of molecular variance (AMOVA) as implemented in ARLEQUIN. Two-way AMOVA was conducted to estimate the variation associated with the differences among groups of populations, populations within groups and individuals within populations. For the comparison, one-way AMOVA was conducted by considering each group of populations separately to compare the distribution of genetic variation among vs within the populations within groups. GenAlEx was used to compute and test the statistical significance of Fi-statistics using a nonparametric approach based on 1000 random permutations of haplotypes.

The levels of differentiation between all populations and among populations within the assumed groups of populations (i.e. *Rvi6*-group, *Rvi1*-group, *Rvi17*-group, *Rvi0*-group or general non-*Rvi6*-group) were estimated using Weir and Cockerham’s pairwise estimator Ɵ [37], which is an analogue of Wright’s F_ST_, and its statistical significance was assessed using a permutation test (1000 permutations) implemented in the GENETIX [38] programme.

Nei’s unbiased [33] pairwise estimates of genetic distance between the assumed groups of populations were calculated using GenAlEx 6.5.

Pairwise gene flow (*Nm*) was estimated in GenAlEx for all populations, where *Nm* < 1 indicated low levels of gene flow, *Nm* = 1 indicated that the drift effects were exactly counterbalanced by the effects of gene flow so that populations neither diverged nor converged, and *Nm* > 1 suggested high levels of gene flow [39].

### Multilocus linkage equilibrium analysis

Allele associations among different loci were examined using a generalized measure of multilocus linkage equilibrium (*r*BAR_*d*_) [40, 41], which is a modification of the index of association (*I*_*A*_) and is independent from the number of loci included in the analysis. The null hypothesis of the random association among alleles at different loci (*r*BAR_*d*_ = 0), indicating random mating, was tested across all assumed groups of populations using the programme MULTILOCUS 1.2.2. [41]. The observed value of the statistic was compared to that obtained after 1000 randomizations to simulate recombination.

### Isolation by distance

To test whether the genetic structures of the populations were influenced by Wright’s isolation by distance theory, the Mantel test was performed using the GenAlEx and Isolation By Distance Web Service 3.23 [42] programmes. The programmes computed the correlation between the pairwise genetic distance matrix (e.g., linearized F_ST_ by Slatkin or Rouset’s) and the pairwise Euclidean geographical distance matrix, both of which were log or not transformed.

## Results

### Preparation of the dataset and allele frequency estimations in the populations

The single, clear amplicons comprising 11 microsatellite loci were obtained for 633 samples. Clone correction using GenoType software revealed the presence of repeated haplotypes in 8 populations, while the highest clone percentage was observed for the END (*Rvi6*) population (Table 1). After removing clones, the data from 606 individuals belonging to 20 populations (each comprising 25–32 individuals) were analysed. A total of 171 alleles were found across the 11 microsatellite loci, with the number of alleles per locus ranging from 5 for *1aac3b* to 32 for *1tc1g* (mean = 15.6). For the groups of populations, the lowest allelic richness was observed for the *Rvi6*-group (3.563), while the values for the other groups were higher and at a similar level (*Rvi17*-group = 6.527, *Rvi1*-group = 6.275 and *Rvi0*-group = 6.370). The highest value for a population was for the ASI population (from the *Rvi17*-group, Table 1). The frequency of private alleles was the highest in two *Rvi17* populations, ASI and ABR, while the lowest frequency or total absence was observed mostly in the *Rvi6* populations (Table 1). The highest number of alleles per locus was observed for the *1tc1g* locus (32 alleles), while the lowest was observed for the *1aac3b* locus (5 alleles).

### Clustering and assignment methods

**Fig. 2:**
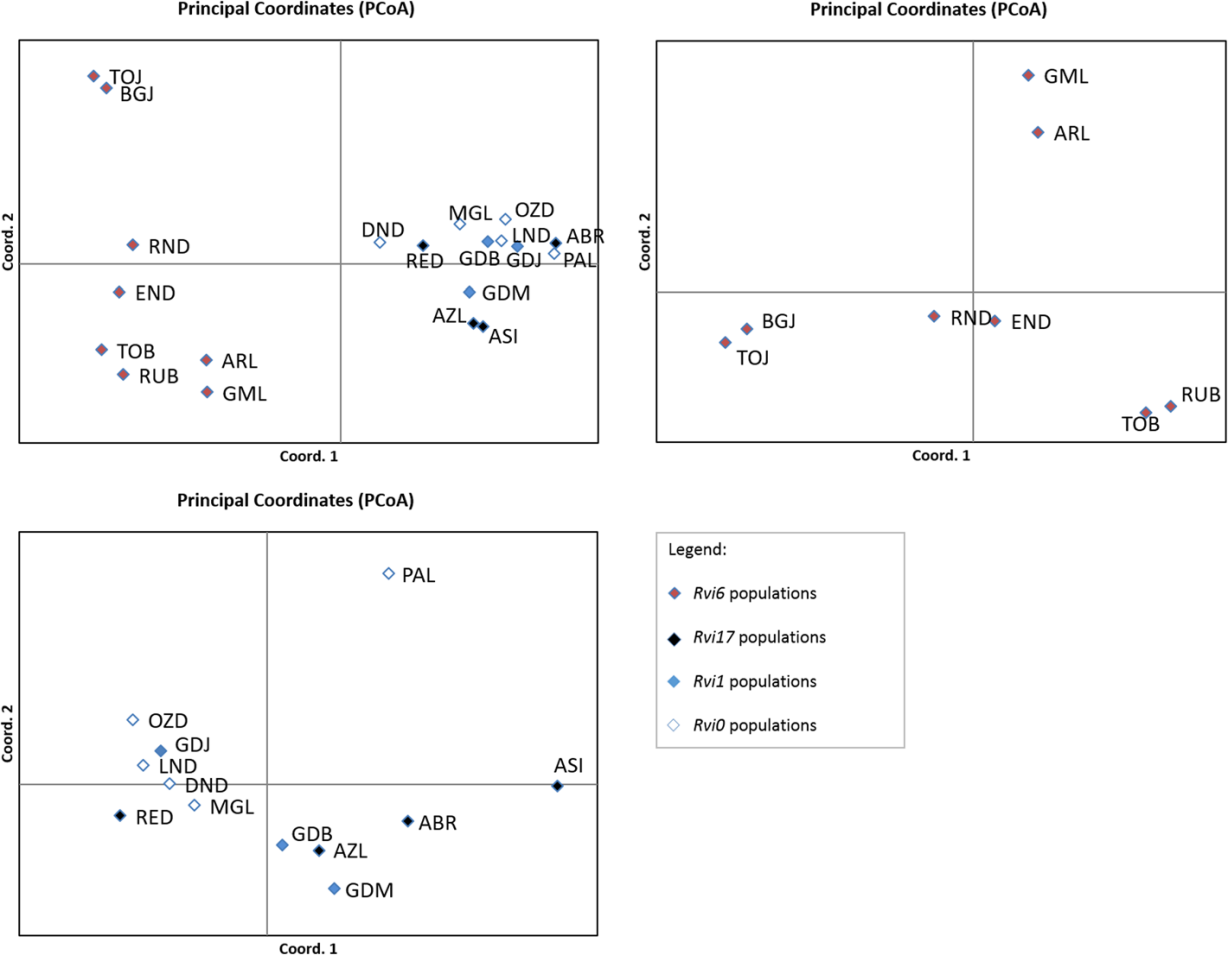
The principal coordinate analysis of a chord distance matrix among (**a**) 20 populations of *V. inaequalis*, (**b**) 8 populations of *V. inaequalis* derived from the *Rvi6* apple cultivar and (**c**) 12 populations of *V. inaequalis* derived from the *Rvi1*, *Rvi17* and ‘*Rvi0*’ cultivars

PCoA analysis separated the *V. inaequalis* populations into two distinct groups, with the first and second principal coordinates accounting for 25.06% and 10.51% of the total variation, respectively. The first principal coordinate separated the populations into two main groups, in which one group contained only the *Rvi6* populations (Fig. 2a). When these groups of populations were tested separately, the first and second principal coordinates divided eight *Rvi6* populations from Lublin, Jeziorsko, Nowy Dwor and Brzeziny according to their geographical origin (Fig. 2b), whereas no spatial structures were observed for the non*-Rvi6* populations (Fig. 2c).

The exclusion-based method implemented in GeneClass produced an accurate assignment rate (the probability that an individual belongs to the reference population within which it was sampled) of 22.8% when all the individuals and populations were tested. Of the *Rvi6*, *Rvi17*, *Rvi1* and *Rvi0*-groups of populations, the highest rate of accurate assignment was observed for the *Rvi6*-group (61.5%), while the lowest rate of accurate assignment was observed for the miscellaneous group comprising populations from cultivars without known apple scab resistance genes (*Rvi0*, 37.2%). Overall, the rate of misassignment (an individual having a genotype that is most likely to occur in a population other than the one in which it was sampled) was high, as many individuals from one host cultivar group tended to be assigned with high probability to all the other host groups, but the individuals from the *Rvi6*-group were mostly assigned to the other populations from this group.

**Fig. 3:**
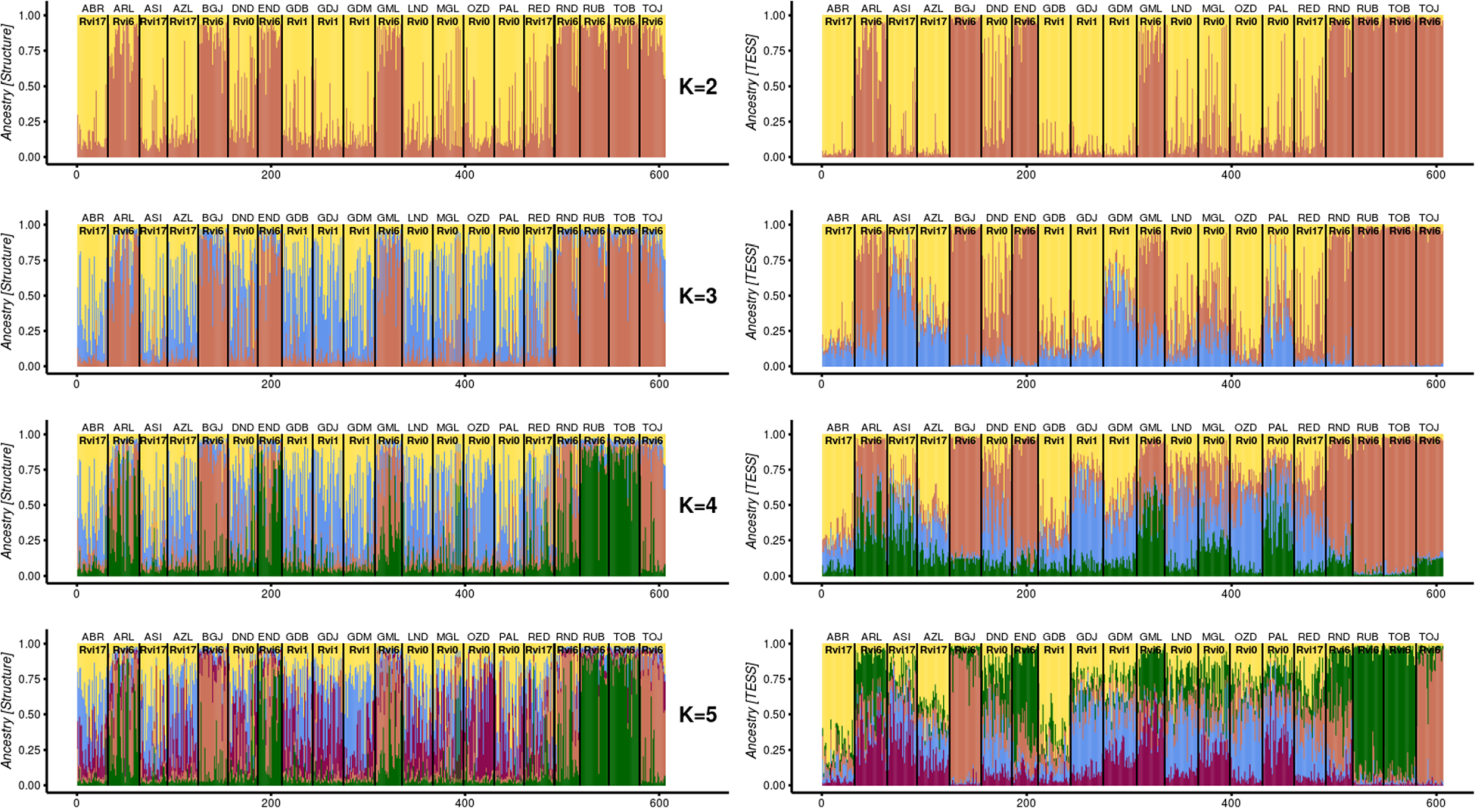
Ancestral proportion of *Venturia inaequalis* haplotypes from the K = 2 to K = 5 clusters inferred with STRUCTURE 2.3.4 and TESS 2.3.1 software on the left and right, respectively. In total, 606 strains are grouped according to the origin and cultivar of their host plant. Each vertical bar representing an individual is partitioned into K clusters. The dominant colour for each population indicates its affiliation to the inferred cluster

**Fig. 4:**
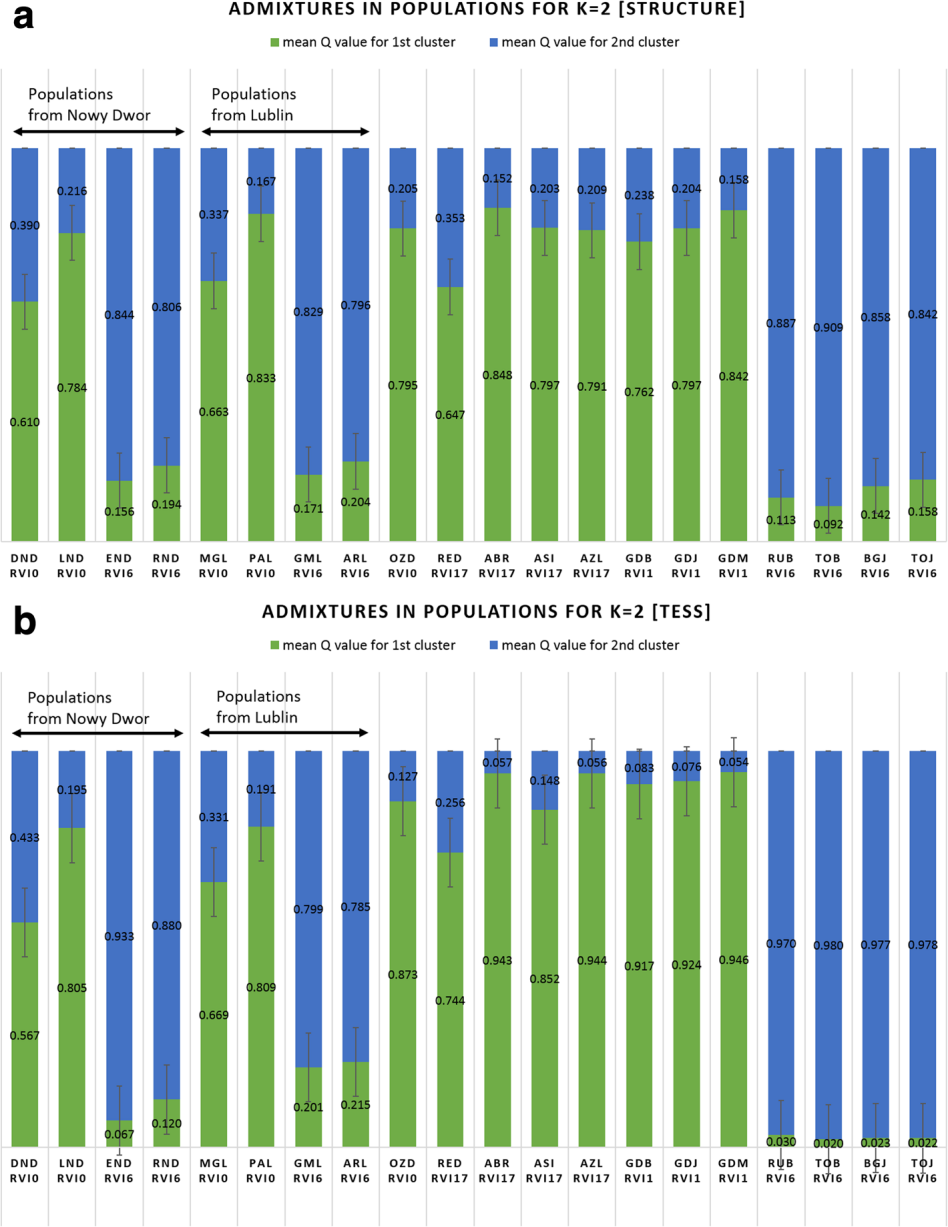
Barplot showing the average mean Q values for 20 *V. inaequalis* populations, representing the admixture between two main inferred clusters: one comprising *Rvi6* and the other *Rvi6*-avirulent populations. Each bar contains the Q values calculated by a) STRUCTURE 2.3.4 or b) TESS 2.3.1 software

To estimate the most likely number of ancestral populations (K), individual-based Bayesian clustering analyses were performed using the STRUCTURE and TESS programmes. Using STRUCTURE, we observed the highest Evanno’s ΔK index for K = 4, but this index had low statistical support (ΔK = 14.73) (Additional file 1: Fig. S1, left graph). Using TESS, we determined that the average DIC values for the K values between 2 and 10 clearly gradually decreased from K = 2 to K = 5 and then marginally varied (lowest DIC for K = 7), supporting 5 to 7 clusters (Additional file 1: Fig. S1, right graph). Taken together, we did not find evidence for an ‘optimal K’ number of clusters. To avoid overinterpretation, we elected to interpret a series of admixture plots for K values from 2 to 5 (Fig. 3). At K = 2, both programmes gave very similar results and divided all the individuals according to their virulence towards *Rvi6* hosts. Indeed, most samples collected from the *Rvi6* trees were assigned to a single group, while samples collected from the other trees (*Rvi1*, *Rvi17* and *Rvi0*) were grouped together. In both analyses, the algorithms detected some admixed individuals. From K = 3 to K = 5, STRUCTURE and TESS found evidence of additional groups, but they were irrespective from any other sources of resistances, suggesting that the algorithms actually indicated different levels of substructure within both the *Rvi6* and the non-*Rvi6* cluster. At K = 3, STRUCTURE found support for a substructure within the non-*Rvi6* gene pool, while TESS found a third group within the *Rvi6* cluster that preferentially comprised individuals of populations from Lublin and non-*Rvi6* populations. At K = 4 using STRUCTURE, all the *Rvi6* populations were divided into two clusters that each comprised populations from two distant geographical locations, whereas the rest of the populations were separated to two other clusters. At K = 4, the fourth cluster in TESS (green colour) favoured both *Rvi6* and non-*Rvi6-*virulent populations from Lublin. Individual clustering observed for K = 5 in STRUCTURE was very similar to that at K = 4. Four subgroups among the *Rvi6* populations were distinguished at K = 5 in TESS, corresponding with the geographical origin of the populations, whereas non-*Rvi6* populations were in one heterogeneous cluster, with different levels of admixture for the individuals. For K = 7, indicated as the optimal number of clusters using TESS, some substructures were observed among the *Rvi6* populations. However, generally most individuals were poorly assigned to the inferred clusters (Additional file 2: Fig. S2). Due to some factors that can substantially affect clustering and ancestral population inference, such as effective sample size or linkage between markers, there is a risk that clusters indicated by the Bayesian algorithms may not correspond to real populations [25]. In practice, rigorous estimation of K is a difficult statistical problem, even when all the assumptions of the underlying model are assumed to hold [43]; thus, the clustering results should be interpreted with care. This could be the possible explanation for why the same individuals were divided into various numbers of optimal clusters dependent upon the method used, although this had only a low level of support. In spite of that, all cluster analyses clearly indicated the existence of two main clusters, one comprising *Rvi6-*virulent individuals and the other comprising *Rvi6*-avirulent individuals. Interestingly, at K = 2, a higher average level of admixture was observed in the DND and MGL populations, which were collected in the orchards in Nowy Dwor and Lublin, respectively, with co-occurrence of the *Rvi6* and non*-Rvi6* cultivars (Fig. 4 a and b).

In cluster analysis conducted in GDA, on UPGMA dendrogram three clades were designated: two with only *Rvi6* populations and one with the rest of the populations, while on NJ dendrogram all *Rvi6* populations were in one clade, and other populations were scattered, although the support for the nodes was low (< 50) (Additional file 3: Fig. S3).

### AMOVA

The two main groups of populations revealed in clustering methods (one containing only *Rvi6* populations and the other containing the remaining non-*Rvi6* populations were analysed using two-way AMOVA. Analysis of molecular variance for the two groups revealed that differentiation was low, albeit significant, at all hierarchical levels (among populations in a group F_SC_ = 0.076, among groups F_CT_ = 0.076, among total genetic distance between all populations F_ST_ = 0.1123). The largest genetic variation was partitioned between individuals within populations (85%), while variation among groups (7.71%) was comparable to those among populations within groups (6.97%). When each group of populations was considered separately, the largest genetic variation was again partitioned between individuals within populations (84.0% for the *Rvi6*-group and 96.5% for the non-*Rvi6*-group), but the variation among populations was much higher in the *Rvi6*-group (16%) than in the non-*Rvi6*-group (3.50%). The Fi-test conducted using GenAlEx confirmed a significant difference between these two groups (Φ’_PT_ = 0.013, *p* = 0.001).

### Gene diversity and pairwise estimates among populations and groups of populations

The overall unbiased gene diversity (*H*_*D*_) calculated for all 20 populations was 0.613. The highest values were observed in ASI (0.762) from the *Rvi17* populations and PAL (0.740) from the *Rvi0* populations, and the lowest values were observed in BGJ (0.413) and GML (0.431) from the *Rvi6* populations (Table 1). When the groups of populations were tested, assumed according their virulence towards the *Rvi6*, *Rvi1*, *Rvi17* and *Rvi0* hosts, the lowest mean *H*_*D*_ value (0.501) from all the analysed population groups was noted in the *Rvi6*-group, while mean values for the other groups were determined to be 0.684 (*Rvi17*), 0.678 (*Rvi1*) and 0.698 (*Rvi0*).

Nei’s unbiased genetic pairwise estimates identity or distance calculates the genetic identity or diversity between populations across all loci simultaneously with the assumption that differences can arise due to both mutation and genetic drift. The values obtained for the two assumed groups, *Rvi6* and non-*Rvi6,* were 0.148 and 0.862, respectively. When four assumed groups of populations were compared in pairs (*Rvi6* vs *Rvi1*, *Rvi6* vs *Rvi17*…etc.), the highest values of genetic distance between the groups were always observed for pairs comprising the *Rvi6*-group (0.141–0.173), while the genetic distances observed for the other pairs were much lower (0.020–0.027) (Additional file 4: Table S1).

F_ST_ values can range from 0 for identical populations (no population differentiation) to 1 for populations sharing no common alleles (complete differentiation in which populations are fixed for different alleles) [44]. Pairwise estimates of differentiation (F_ST_ or Ɵ) calculated among the 20 populations studied indicated medium (F_ST_ = 0.10772; *p* < 0.001) total differentiation among the populations. The pairwise estimates of F_ST_ values computed for all the pairs of populations were the lowest among the *Rvi6* populations when these populations came from the same orchard. Interestingly, the distances between the *Rvi6* and non-*Rvi6* populations from the same orchard (Lublin and Nowy Dwor) were usually lower than those between the ARL & GML, END & RND *Rvi6* populations and the *Rvi6* populations from other orchards (Additional file 5: Table S2). Apart from these exceptions, the *Rvi6* populations generally shared low F_ST_ values with other *Rvi6* populations, while populations from other groups shared the lowest F_ST_ values with populations from various groups but never with populations from the *Rvi6*-group. When four groups of populations were estimated in pairs, the F_ST_ values for pairs including the *Rvi6* populations were always higher than those values shared between the *Rvi1*, *Rvi17* and *Rvi0* groups (Table 2). When F_ST_ values among populations within groups were estimated, the highest genetic distance was observed for the *Rvi6* group (F_ST_ = 0.1570), while the values in the other groups were low (*Rvi17* = 0.0389, *Rvi0* = 0.0279, *Rvi1* = 0.0393).

**Table 2:** Measure of pairwise comparisons of genetic distance (F_ST_, above diagonal) and gene flow (*Nm*, below diagonal) estimated for four assumed groups of populations of *V. inaequalis*

Group	*Rvi6*	*Rvi17*	*Rvi1*	*Rvi0*
*Rvi6*	...	0.08508	0.09155	0.07606
*Rvi17*	1.868	...	0.00940	0.00818
*Rvi1*	1.97	8.495	...	0.01127
*Rvi0*	2.122	7.063	8.174	...

The observed *H*_*D*_ and Nei’s values and the F_ST_ estimates clearly showed that the *Rvi6* populations were genetically closer to other *Rvi6* populations, especially those derived from the same orchard. However, they were subdivided within the whole *Rvi6*-group. Moreover, the *Rvi6* populations were clearly distanced from the non-*Rvi6* populations that came from other orchards, which were in turn more closely related to each other. An exception was observed between the *Rvi6* and non-*Rvi6* populations occurring in sympatry, for which the F_ST_ values indicated very close (Lublin) to the closest (Nowy Dwor) genetic relationship among the populations.

These data are consistent with those from the clustering and assignment methods and explain the differences among the *Rvi6* and non-*Rvi6* populations, the substructures within the *Rvi6-*group, and the genetic heterogeneity among the *Rvi17*, *Rvi1* and *Rvi0* groups of populations.

Gene flow, considered as a movement of genes, can be detected in populations by observing the movement or dispersal of whole organisms or genomes from one population to another [45]. Pairwise estimates of gene flow (*Nm*) between all populations (Additional file 6: Table S3) and groups of populations (Table 2) were always higher than 1, suggesting gene flow between populations, although with different intensities. The highest values were observed among pairs of the *Rvi6* populations (2.321–33.836, mean 11.960), derived from the same orchard. The mean *Nm* value between the *Rvi6*-group and the non-*Rvi6*-group was 2.441, while the mean values between the other groups (*Rvi17*, *Rvi1* and *Rvi0*) were between 7.063 and 8.495. These data may suggest that gene flow between the *Rvi6* and non-*Rvi6* populations can be limited but not excluded.

### Random mating hypothesis testing

The index of association relies on the variance of genetic distances between pairs of individuals. Low variance occurs in a population with free recombination across loci, while high variance occurs when recombination is rare [46]. The null hypothesis of random alleles association (*r*BAR_*d*_ = 0) from random mating can be rejected only when the value is significantly different from zero after 1000 randomizations. The null hypothesis of random mating could not be rejected for any of the tested groups of populations (i.e. *Rvi6*, *Rvi1*, *Rvi17*, *Rvi0* groups) or for the whole non-*Rvi6*-group. Thus, we assumed that among the populations within the groups, free recombination was possible. When the groups were tested in pairs, the null hypothesis was rejected only in pairs with the *Rvi6*-group, indicating that random mating did not occur between the *Rvi6* and the non-*Rvi6* populations. However, when the *Rvi6* and non-*Rvi6* populations sampled from the same orchard (Lublin or Nowy Dwor) were tested, the null hypothesis of random mating between these populations could not be rejected, indicating that random mating was possible between the sympatric populations.

### Isolation by distance

Because the regression correlation analysis between genetic and geographic distances among all populations was not significant (*p* > 0.05), the genetic variation among populations cannot be explained by the geographical origin of the populations. When the *Rvi6* and non-*Rvi6* groups of populations were tested separately, the same tendency was observed. This indicates that genetic population structures are not influenced by the geographic origin of the populations.

## Discussion

Resistance genes introgressed by humans to crops are generally broken down soon after they are deployed into the agroecosystem as a result of the emergence of a virulent pathogen population. For quickly evolving organisms, such as plant pathogens, new adaptations occur easily in just a few generations after an environmental change [47]. New virulent strains are generally assumed to originate from new de novo mutations at the avirulent locus from populations already present in agroecosystems. Pathogens can rapidly adapt to changes since they generally have higher fecundity, larger effective sizes and shorter generation times than their hosts [48]. As an alternative scenario of virulence emergence, Leroy et al. suggested that virulent strains can be selected from pre-existing virulent populations in non-agricultural habitats. The latter scenario of adaptation from standing genetic variation can be favoured, especially when pathogens infect various non-agricultural hosts, including weeds and wild plants growing near cultivated plants or those widely spread by humans. All these factors can explain the rapid breakdown of resistance genes in agroecosystems [49], including the *Rvi6* gene [13].

### Genetic uniqueness of *V. Iniaequalis* populations derived from *Rvi6* hosts

The pathosystem *V. inaequalis - Malus* spp. was found to be suitable for studying both gene flow and host adaptation processes, which are expected to shape the genetic structure of pathogen populations overall [12]. The previous findings of *V. inaequalis* populations overcoming *Rvi6* gene-related resistance in apple cultivars showed that newly emerging fungal populations were genetically different from populations from plants without *Rvi6* and demonstrated the clonal structure [9, 10, 15]. The results obtained in our study also indicate the genetic distinctness of *Rvi6*-virulent populations from the populations infecting *Rvi17*-, *Rvi1*- and cultivars without any known *R* genes. First, based on results obtained in the clustering methods that implemented various algorithms (Bayesian inference, PCoA and distance matrix-based), the division of all populations into two main groups was evident. The *Rvi6* populations formed a separate cluster or clusters depending on the algorithm used, which did not contain any non-*Rvi6* populations. However, in each *Rvi6* or non-*Rvi6* cluster, individuals showing admixed ancestry from both clusters were observed (STRUCTURE, TESS). In parallel, the probability of assignment of *Rvi6* individuals to non-*Rvi6* populations was low (less than or equal to 10%). Second, the *1tc1g* locus, which had the highest number of alleles in the *Rvi6* populations, was represented by only 7 alleles out of a total of 32 alleles observed for this locus. However, these alleles were also present in almost all the other populations. Since the *1tc1g* locus is considered to be linked to the *AvrRvi6* locus of *V. inaequalis* [11], the observed tendency for a reduced number of alleles at this locus in *Rvi6*-virulent populations is consistent with the genomic signature of the selective sweeps hypothesis [50]. This hypothesis states that when a selectively favourable mutation occurs in a population and subsequently becomes fixed in that population, selective sweeps change the allelic frequency at closely - linked loci, which can result in a local reduction of genomic variation. Moreover, the observed reduced diversity in multiple regions of the genome is also consistent with other studies [9, 11]. The fact that the *Rvi6* populations exhibited the lowest allelic richness, frequency of private alleles and mean genetic diversity across the 11 loci and the highest clonal fraction show that the genetic structure of these populations is the response to a main apple resistance gene breakdown. On the one hand, the observed reduced genetic diversity at the population level fits the primary concept of gene-for-gene interaction [3], which can be realized by the ‘mutation-to-virulence’ scenario in which a molecular event occurring in a compatible avirulence gene in a single individual leads to the selection of a new subpopulation and the emergence of virulence within the agroecosystem. On the other hand, the second scenario considered here is the possibility of the migration of a few virulent individuals from pre-existing virulent populations in non-agricultural reservoirs. In such case, maintenance of a high population differentiation in several genomic regions is expected between the ‘native’ and immigrating populations because of population divergence in allopatry, isolation for long periods and current restricted gene flow between them [49]. Based on the observed reduced genomic diversity as well as the results of the assignment and clustering analyses, Guérin and co-workers [11] raised the hypothesis that the origin of *Rvi6-*virulent individuals can be external, and these individuals did not emerge from the selection of a mutated *avrRvi6* allele in any of the sampled non-*Rvi6* populations. They assumed that the *Rvi6* populations derived from a single ancestral population after a substantial reduction of variability caused by a demographic bottleneck, and the current *Rvi6* populations were founded by just a few related individuals. Lemaire et al. used demographic modelling to demonstrate that the two distinct *Rvi6* and non-*Rvi6* lineages diverged several thousands of generations ago without subsequent detectable gene flow [13]. Their studies proved the hypothesis that populations infecting *Rvi6* varieties emerged from pre-existing populations present on the non-agricultural progenitor *M. floribunda*, donor of *Rvi6* gene, thus supporting the second scenario regarding the migration of virulence between ecosystems. Secondary contact between subpopulations is currently observed in some European apple tree orchards, representing the opportunity for adaptive pathogenic evolution [14], including Poland.

Whereas the overall level of genetic polymorphism was low in the *Rvi6* populations, we observed that the level of variation among these populations was almost five times higher than that within the non-*Rvi6* group (AMOVA). Moreover, the *Rvi6*-group exhibited the highest F_ST_ values among groups, within itself, suggesting that *Rvi6* populations are genetically subdivided. The lack of correlation between the genetic and geographic distances for the populations indicated that the genetic structure of the populations was not influenced by their geographic origin. However, some grouping related to geographic origin was observed in the segregation performed by the TESS, STRUCTURE (for K = 5) and PCoA algorithms. The strong evidence of substructures among the *Rvi6* populations can be explained by the hypothesis assuming several recent instances of migration of virulent individuals from the non-agricultural to the agricultural pathosystem and then undergoing further distribution by rare, long-distance dispersal events. The orchards with *Rvi6* cultivars are not very common in Poland; the distance between the examined neighbouring orchards containing *Rvi6-*cultivars is in range from 50 to 220 km, while the experimental orchard with various mono- and poli- *R*-gene apple cultivation is located in Dabrowice, in a distance from 18 to 220 km to the nearest commercial orchards containing *Rvi6* cultivars. Therefore, we assume that there is a limited exchange of virulent strains between orchards, and the emergence of disease in *Rvi6* hosts in previous decade is most likely a result of human activities regarding the transportation of infected apple tree leaves over long distances. In our study, it was possible to observe a structure related to the host only for the *Rvi6* populations that was maintained over years because of impeded gene flow between immigrants and local populations. In the non-*Rvi6* populations, neither significant population structure nor correlation between the assigned cluster and the host cultivars were detected. Even if the geographic distance between the non-*Rvi6* populations was significant, they shared common genotypes. Here, free dispersal of the fungus through large apple tree areas both by wind or human-mediated actions together with gene flow lead to genetic homogeneity among populations within regions. Similarly, the lack of substructure linked to different hosts was observed for the populations derived from various host in single orchards [9, 12]. The findings of higher allelic and private allelic richness as well as higher genetic diversity in the non-*Rvi6* populations compared to the *Rvi6* populations together with the small or absent clonal fractions support the hypothesis that non-*Rvi6* populations are evolutionarily older than the *Rvi6* populations [51]. The other possibility is that the *Rvi6* individuals observed currently in the agroecosystem are only a small representation of the whole *Rvi6*-virulent population.

### Genetic distinctness of *V. Inaequalis* populations derived from *Rvi6* and non-*Rvi6* hosts

The results obtained in this study revealed clear genetic distinctness among populations that were virulent or non-virulent towards the *Rvi6* apple tree cultivars, which was detectable in most of the sampled orchards. The assignment test performed using GeneClass showed that the *Rvi17*, *Rvi1* and *Rvi0* groups exchanged individuals quite randomly, causing relatively low genetic distance between these groups as detected by other programmes, whereas the individuals from the *Rvi6*-group were frequently assigned to other populations within the same group. Genetic distance, inferred from Nei’s unbiased measure of genetic distance and F_ST_ values, was always the highest between the *Rvi6*-group and other groups of populations. The *Rvi6* populations were clearly distanced from non-*Rvi6* populations coming from other orchards and genetically closer to other *Rvi6* populations, especially those derived from the same orchard. Moreover, the F_ST_ values estimated for the groups was the highest within the *Rvi6*-group, indicating higher differentiation between populations in the *Rvi6*-group than between populations in each of the non-*Rvi6* groups, which agrees with the observed genetic subgroups among the *Rvi6* populations. Conversely, the non-*Rvi6* populations, even those derived from distant orchards, were genetically mixed but with an overall low level of genetic differentiation and variation. The random mating hypothesis testing showed that random mating was not possible between populations from the *Rvi6*-group and other groups, but was possible among individuals from the *Rvi17*, *Rvi1* and *Rvi0* groups. This probably explains why the clustering programmes assumed that non-*Rvi6* populations originated from a single panmictic population. Our finding regarding random mating within the *Rvi6*-group differs from that of Guérin et al.; their linkage disequilibrium testing results were not consistent with random mating among *Rvi6* populations [11].

The high structuring effect of the presence or absence of a single resistance gene in host plants, *Rvi6* in this study, led to a split of the pathogen population into two subpopulations. This phenomenon was previously observed in commercial orchards between infected *M x domestica* cultivars carrying or not carrying the *Rvi6* gene on large scale [9, 11, 13]. Reported studies demonstrated the lack of gene flow between individuals from *Rvi6* and non-*Rvi6* hosts, what was observed between populations sampled at multiple sites on multiple host species and varieties [13], but also between sympatric populations [52]. Gladieux et al. [10] confirmed very high host specificity of *V. inaequalis* strains virulent toward *Rvi6* cultivars, that correlated with strong selection against immigrants in *V. inaequalis* populations. The strong genetic differentiation between the two populations may indicate, that the pathogenic capability of *Rvi6-*virulent individuals on non-*Rvi6* host and their ability to compete with *Rvi6-*avirulent individuals on non-*Rvi6* hosts may have been reduced. This in turn reduces the probability of meeting and mating within the host, resulting in detectable reproductive isolation between sympatric populations [10]. Therefore, the population structure between *Rvi6* and non-*Rvi6* populations is usually maintained in agroecosystems, even when *Rvi6* and non-*Rvi6* cultivars are planted in the same orchard [10, 12].

On the other hand, the genetic distinctness between *Rvi6* and non-*Rvi6* populations was broken between the sympatric populations derived from orchards located in Nowy Dwor and Lublin, which was supported by five observations. First, the evidence of admixture between the two types of populations, expressed as *Rvi6*-virulent and avirulent individuals grouped in one cluster, was detected using TESS. This was most clear for populations from Lublin (K = 4, green colour) and was slightly detected for populations from Nowy Dwor (K = 5, all sympatric populations have the admixture of green colour, which is ‘dedicated’ to *Rvi6* populations on this plot, Fig. 3). Among them, the DND (Nowy Dwor) and MGL (Lublin) populations showed the highest average level of admixture between *Rvi6* and non-*Rvi6* populations from all non-*Rvi6* populations. Second, the F_ST_ pairwise estimates of differentiation were usually lower between the *Rvi6* and non-*Rvi6* populations sampled from the same orchard than between these populations from other orchards. Third, the misassignment rate in the populations in Nowy Dwor and Lublin orchards was high, revealing that individuals were assigned with high probability to more than one population. Fourth, the null random mating hypothesis could not be rejected for sympatric populations, neither in Lublin nor in Nowy Dwor, indicating that random mating was possible between the sympatric populations. Moreover, comparisons of the general pairwise gene flow estimates between the *Rvi6* and non-*Rvi6* groups revealed that gene flow was detectable, although the values were low. These results allowed us to assume that virulent strains towards *Rvi6* cultivars could infect non-*Rvi6* cultivars and that they coexisted on host trees without the *Rvi6* gene during the sampling time in Nowy Dwor and Lublin.

The coexistence of strains of two types of virulence on one host was reported previously in glasshouse conditions [10], but evidence of gene flow between the populations has thus far only been observed in one experimental dessert apple orchard [14]. To our knowledge, this study is the first report of secondary contact followed by gene flow between strains infecting *Rvi6* and non-*Rvi6* cultivars in commercial organic orchards in Poland that were sampled from various *Malus* x *domestica* hosts. The coexistence of two strains with different virulence patterns that diverged a long time ago and were then isolated from one another by ecological barriers on one apple tree creates the possibility for mating and recombination. Expected recombination between the two populations might promote the evolution of aggressiveness, a major component of fitness in fungal pathogens, by combining aggressiveness factors from each type of population in hybrids [14, 47]. This helps hybrids adapt to monogenic resistant hosts that are newly introduced into the agroecosystem. The consequences of the rapid evolution of *V. inaequalis* that arose from secondary contact need to be investigated by tracking not only aggressiveness but also the different stages of the life cycle of the pathogens to explore whether admixed individuals show higher or lower fitness than their ancestors. The discovery of new *Rvi6-*virulent populations and the evidence of gene flow occurring between the new and the existing ‘native’ populations in organic orchards in Poland and other European countries is important from an epidemiological aspect since *Rvi6* cultivars are not protected in these regions by fungicide sprays, thus the virulent populations and possible hybrids are maintained in the ecosystem. Planting fully susceptible cultivars in the same orchard facilitates the breakdown of ecological reproductive isolation between agricultural and non-agricultural pathogen populations [14], which can seriously influence sustainable disease management.

The structure of *Rvi6-*virulent populations agrees with the hypothesis that gene-for-gene relationships exert a structuring effect for the genetic shape of compatible fungal populations. We expected to observe the same effect of main *R* gene of other host plants on *V. inaequalis* populations, however, this was only evident in newly emerging virulent populations and not for those that have been present for a long time in the pathosystem. Considering the possibility that the two populations - virulent and avirulent to *Rvi6* - can infect the same hosts and mate, borders between them are expected to melt, especially in the orchards in which these populations occur in sympatry. Based on the observations of migrants and F1 hybrids at the European scale and hybrid swarms in some orchards, Lemaire et al. and Leroy et al. concluded that the immigrant inviability barrier is eroding because of possible mating events between *Rvi6* and non-*Rvi6* strains on host varieties susceptible to both kind of pathogens [13, 14]. Assuming that the gene flow between divergent individuals might promote evolution of aggressiveness in pathogen populations [14], the risk of spread of hybrids with transgressive traits over other apple growing regions cannot be excluded.

## Conclusions

Population structure analysis of *V. inaequalis* populations sampled from several orchards across Poland concluded that there are two main population groups, one infecting *Rvi6* cultivars and one infecting cultivars that have other sources of resistance or no known resistance at all*.* Based on the Bayesian analysis of 20 fungal populations and distance based methods, we also found support for subgroups within the *Rvi6* populations. This is consistent with several independent introduction events (i.e. different long-distance dispersals from wild *Rvi6* host species), which were most likely enhanced by human actions instead of selection of individuals from any of the sampled non-*Rvi6* populations. The genetic structure of the *Rvi6*-virulent populations being clearly distinct from that of the non-*Rvi6* populations is recognized as the host adaptation effect. Specifically, the selective pressure is exerted by the main apple scab resistance gene (*Rvi6* in this study). However, we did not observe such an effect in the other *V. inaequalis* – apple tree interactions. Gene flow can occur between two divergent populations, leading to the evolution of hybrid swarms, especially when the representatives coexist in one orchard or tree. Such secondary contact may lead to evolutionary and epidemiological changes. Considering the ecological, economic and nutritional importance of apples in temperate countries, especially Poland, monitoring the emergence of new virulence is particularly important to allow for effective preventive strategies.

## Additional files

Additional file 1: **Fig. S1.** Criteria used to determine the appropriate cluster solution. The left graph shows the delta K plot from the STRUCTURE 2.3.4. runs based on mean (|L``(K)|)/sd(L(K)) values, and the right graph shows the plot of the mean DIC value of each TESS 2.3.1. cluster solution. Plot lines were added to help visualize trends. (JPEG 49 kb)

Additional file 2: **Fig. S2.** Ancestral proportion of *Venturia inaequalis* haplotypes for the K = 7 clusters inferred with TESS 2.3.1 software. In total, 606 strains are grouped according to the origin and cultivar of their host plant. Each haplotype is represented by a vertical line partitioned into seven segments. The dominant colour for each population indicates its affiliation to the inferred clusters. (TIFF 706 kb)

Additional file 3: **Fig. S3.** a) Unweighted pair group method with arithmetic mean (UPGMA) and b) Neighbour-joining(NJ) dendrograms based on Nei’s genetic distance [33] between 20 *V. inaequalis* populations, genotyped within 11 microsatellite loci. Numbers at major nodes indicate the percentage of times the cluster to the right of the branch occurred among the sample of 1000-bootstrap-generated dendrograms. (TIFF 3745 kb)

Additional file 4: **Table S1.** Measure of the pairwise comparisons of Nei’s unbiased genetic distance (below the diagonal) and identity (above the diagonal) estimated for the four assumed groups of *V. inaequalis* populations. (XLSX 9 kb)

Additional file 5: **Table S2.** Measure of the pairwise comparisons of genetic distance (F_ST_) estimated for 20 *V. inaequalis* populations. (XLSX 11 kb)

Additional file 6: **Table S3.** Measure of the pairwise comparisons of gene flow (*Nm*) estimated for 20 *V. inaequalis* populations. (XLSX 11 kb)

## References

[1] MacHardy WE (1996). Apple scab: biology, epidemiology and management.

[2] Tenzer I, Gessler C (1999). Genetic diversity of *Venturia inaequalis* across Europe. Eur J Plant Pathol.

[3] Flor HH (1971). Current status of the gene-for-gene concept. Annu Rev Phytopathol.

[4] Bus VG, Rikkerink EH, Caffier V, Durel CE, Plummer KM (2011). Revision of the nomenclature of the differential host-pathogen interactions of *Venturia inaequalis* and *Malus*. Annu Rev Phytopathol.

[5] Parisi L, Lespinasse Y, Guillaumes J, Krüger J (1993). A new race of *Venturia inaequalis* virulent to apples with resistance due to the Vf gene. Phytopathology.

[6] Roberts AL, Crute IR (1994). Apple scab resistance from *Malus floribunda* 821 (Vf) is rendered ineffective by isolates of *Venturia inaequalis* from *Malus floribunda*. Nor J Agric Sci.

[7] Parisi L, Laurens F, Didelot F, Evans K, Fischer C, Fouillet V, Gennari F, Kemp H, Lateur M, Patocchi A, Schouten HJ, Tsipouridis C (2006). Geographical distribution of *Venturia inaequalis* strains virulent to the Vf gene in Europe. IOBC WPRS BULLETIN.

[8] Masny S (2017). Occurrence of *Venturia inaequalis* races in Poland able to overcome specific apple scab resistance genes. Eur J Plant Pathol.

[9] Guérin F, Le Cam B (2004). Breakdown of the scab resistance gene Vf in apple leads to a founder effect in populations of the fungal pathogen *Venturia inaequalis*. Phytopathology.

[10] Gladieux P, Guérin F, Giraud T, Caffier V, Lemaire C, Parisi L, Didelot F, Le Cam B (2011). Emergence of novel fungal pathogens by ecological speciation: importance of the reduced viability of immigrants. Mol Ecol.

[11] Guérin F, Gladieux P, Le Cam B (2007). Origin and colonization history of newly virulent strains of the phytopathogenic fungus *Venturia inaequalis*. Fungal Genet Biol.

[12] Leroy T, Lemaire C, Dunemann F, Le Cam B (2013). The genetic structure of a *Venturia inaequalis* population in a heterogeneous host population composed of different *Malus* species. BMC Evol Biol.

[13] Lemaire C, De Gracia M, Leroy T, Michalecka M, Lindhard-Pedersen H, Guérin F, Gladieux P, Le Cam B (2016). Emergence of new virulent populations of apple scab from nonagricultural disease reservoirs. New Phytol.

[14] Leroy T, Caffier V (2016). Celton J M, anger N, Durel C E, Lemaire C, le cam B: when virulence originates from nonagricultural hosts: evolutionary and epidemiological consequences of introgressions following secondary contacts in *Venturia inaequalis*. New Phytol.

[15] Guérin F, Franck P, Loiseau A, Devaux M, Le Cam B (2004). Isolation of 21 new polymorphic microsatellite loci in the phytopathogenic fungus *Venturia inaequalis*. Mol Ecol Notes.

[16] Ebrahimi L, Fotuhifar KB, Nikkhah MJ, Naghavi MR, Baisakh N (2016). Population genetic structure of apple scab (*Venturia inaequalis* (Cooke) G. Winter) in Iran. PLoS One.

[17] Tenzer I (1999). Degli Ivanissevich S, Morgante M, Gessler C: identification of microsatellites markers and their application to population genetics of *Venturia inaequalis*. Phytopathology.

[18] Meirmans PG, Van Tienderen PH (2004). GENOTYPE and GENODIVE: two programs for the analysis of genetic diversity of asexual organisms. Mol Ecol Notes.

[19] Cavalli-Sforza LL, Edwards AW (1967). Phylogenetic analysis. Models and estimation procedures. Am J Hum Genet.

[20] Dieringer D, Schlötterer C (2003). Microsatellite analyser (MSA): a platform independent analysis tool for large microsatellite data sets. Mol Ecol Notes.

[21] Peakall R, Smouse PE (2012). GenAlEx 6.5: genetic analysis in excel. Population genetic software for teaching and research – an update. Bioinformatics.

[22] Piry S, Alapetite A, Cornuet J-M, Paetkau D, Baudouin L, Estoup A (2004). GENECLASS2: a software for genetic assignment and first-generation migrant detection. J Hered.

[23] Rannala B, Mountain JL (1997). Detecting immigration by using multilocus genotypes. Proc Natl Acad Sci.

[24] Paetkau D, Slade R, Burden M, Estoup A (2004). Genetic assignment methods for the direct, real-time estimation of migration rate using assignment methods: a simulation-based exploration of accuracy and power. Mol Ecol.

[25] Pritchard JK, Stephens M, Donnelly P (2000). Inference of population structure using multilocus genotype data. Genetics.

[26] Evanno G, Regnaut S, Goudet J (2005). Detecting the number of clusters of individuals using the software STRUCTURE: a simulation study. Mol Ecol.

[27] Kopelman NM, Mayzel J, Jakobsson M, Rosenberg NA, Mayrose I (2015). Clumpak: a program for identifying clustering modes and packaging population structure inferences across K. Mol Ecol Resour.

[28] Francois O, Ancelet S, Guillot G (2006). Bayesian clustering using hidden Markov random fields in spatial population genetics. Genetics.

[29] Chen C, Durand E, Forbes F, François O (2007). Bayesian clustering algorithms ascertaining spatial population structure: a new computer program and a comparison study. Mol Ecol Notes.

[30] Durand E, Jay F, Gaggiotti OE, Francois O (2009). Spatial inference of admixture proportions and secondary contact zones. Mol Biol Evol.

[31] Jakobsson M, Rosenberg NA (2007). CLUMPP: a cluster matching and permutation program for dealing with label switching and multimodality in analysis of population structure. Bioinformatics.

[32] Lewis PO, Zaykin D. Genetic Data Analysis: Computer program for the analysis of allelic data. Version 1.0 (d16c). 2001. Free program distributed by the authors over the internet from http://lewis.eeb.uconn.edu/lewishome/software.html.

[33] Nei M (1978). Estimation of average heterozygosity and genetic distance from a small number of individuals. Genetics.

[34] Nei M: Molecular evolutionary genetics, 1st Edn edn. New York, USA: Columbia University Press: 1987.

[35] Goudet J (1995). FSTAT (version 1.2): a computer program to calculate F-statistics. J Hered.

[36] Excoffier L, Lischer HE (2010). Arlequin suite ver 3.5: a new series of programs to perform population genetics analyses under Linux and windows. Mol Ecol Resour.

[37] Weir BS, Cockerham CC (1984). Estimating F-statistics for the analysis of population structure. Evolution.

[38] Belkhir K, Borsa P, Chikhi L, Raufaste N, Catch F: GENETIX 4.0. 5.2., software under windows™ for the genetics of the populations. University of Montpellier, Montpellier, France. 2004.

[39] McDermott JM, McDonald BA (1993). Gene flow in plant pathosystems. Annu Rev Phytopathol.

[40] Brown AHD, Feldman MW, Nevo E (1980). Multilocus structure of natural populations of *Hordeum spontaneum*. Genetics.

[41] Agapow PM, Burt A (2001). Indices of multilocus linkage disequilibrium. Mol Ecol Notes.

[42] Jensen JL, Bohonak AJ, Kelley ST. Isolation by distance, web service. BMC Genet. 2005;6(1):13. http://www.bio.sdsu.edu/pub/andy/IBD.html. 10.1186/1471-2156-6-13 PMC1079815 15760479

[43] Falush D, van Dorp L, Lawson D: A tutorial on how (not) to over-interpret STRUCTURE/ADMIXTURE bar plots. BioRxiv. 2016,066431. 10.1101/066431 10.1038/s41467-018-05257-7 PMC6092366 30108219

[44] Hartl DL, Clark AG (1997). Principles of population genetics.

[45] Mallet J. Gene flow. In insect movement: mechanisms and consequences. In: Woiwood IP, Reynolds DR, Thomas CD, editors. Proceedings of a symposium at the Royal Entomological Society. London: Wallingford, UK: CAB International; 2001. p. 337–360.

[46] Taylor JW, Jacobson DJ, Fisher MC (1999). The evolution of asexual fungi: reproduction, speciation and classification. Annu Rev Phytopathol.

[47] McDonald BA, Linde C (2002). Pathogen population genetics, evolutionary potential, and durable resistance. Annu Rev Phytopathol.

[48] REX Consortium (2010). The skill and style to model the evolution of resistance to pesticides and drugs. Evol Appl.

[49] Leroy T, Le Cam B, Lemaire C (2014). When virulence originates from non-agricultural hosts: new insights into plant breeding. Infect Genet Evol.

[50] Smith JM, Haigh J (1974). The hitch-hiking effect of a favourable gene. Genet Res.

[51] Gladieux P (2010). Zhang X G, Róldan-Ruiz I, Caffier V, Leroy T, Devaux M, van Glabeke S, Coart E, le cam B: evolution of the population structure of *Venturia inaequalis*, the apple scab fungus, associated with the domestication of its host. Mol Ecol.

[52] Giraud T, Villaréal LM, Austerlitz F, Le Gac M, Lavigne C (2006). Importance of the life cycle in sympatric host race formation and speciation of pathogens. Phytopathology.

