# Supplementary figures and images for: Population structure of Venturia inaequalis, a causal agent of apple scab, in response to heterogeneous apple tree cultivation
Source: BMC Evol Biol. 2018 Jan 19;18:5. doi: 10.1186/s12862-018-1122-4 (PMC5775622; doi:10.1186/s12862-018-1122-4)

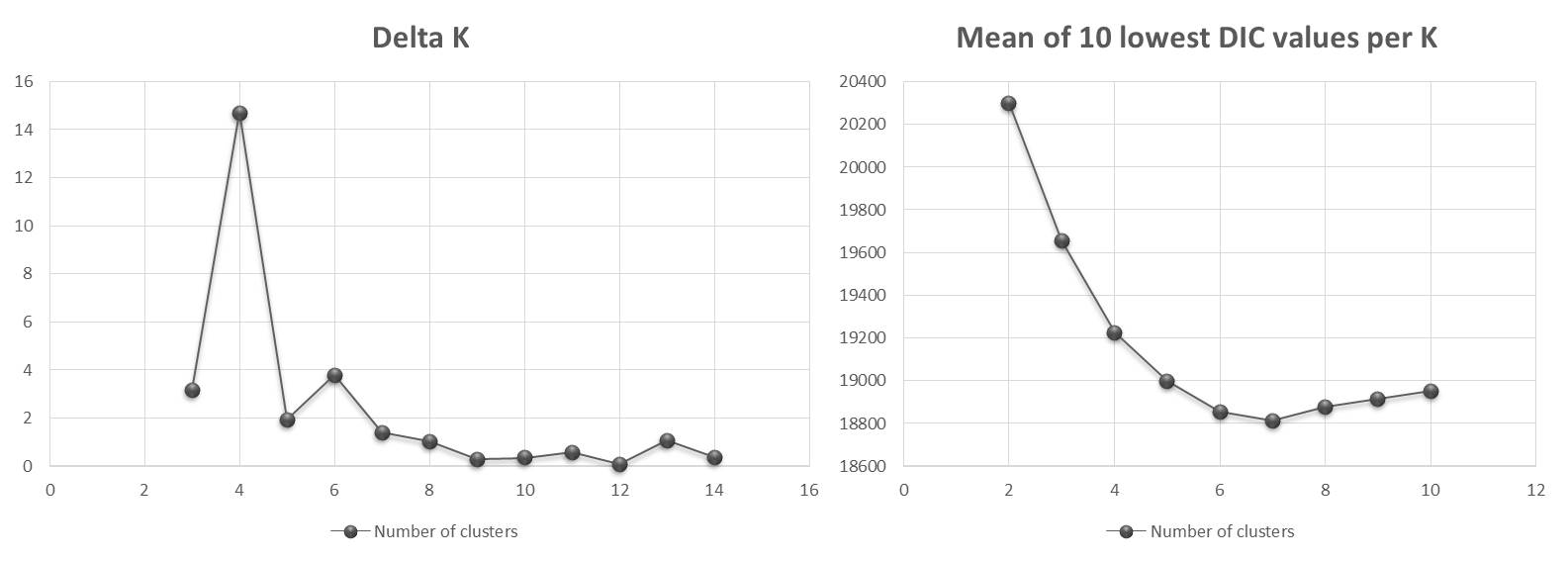

Supplement: Supplementary file 1 — Fig. S1. Criteria used to determine the appropriate cluster solution. The left graph shows the delta K plot from the STRUCTURE 2.3.4. runs based on mean (|L``(K)|)/sd(L(K)) values, and the right graph shows the plot of the mean DIC value of each TESS 2.3.1. cluster solution. Plot lines were added to help visualize trends. (JPEG 49 kb) [file 12862_2018_1122_MOESM1_ESM.jpg]

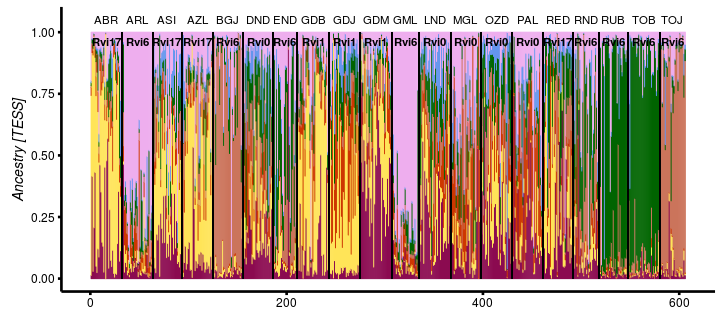

Supplement: Supplementary file 2 — Fig. S2. Ancestral proportion of Venturia inaequalis haplotypes for the K = 7 clusters inferred with TESS 2.3.1 software. In total, 606 strains are grouped according to the origin and cultivar of their host plant. Each haplotype is represented by a vertical line partitioned into seven segments. The dominant colour for each population indicates its affiliation to the inferred clusters. (TIFF 706 kb) [file 12862_2018_1122_MOESM2_ESM.tiff]

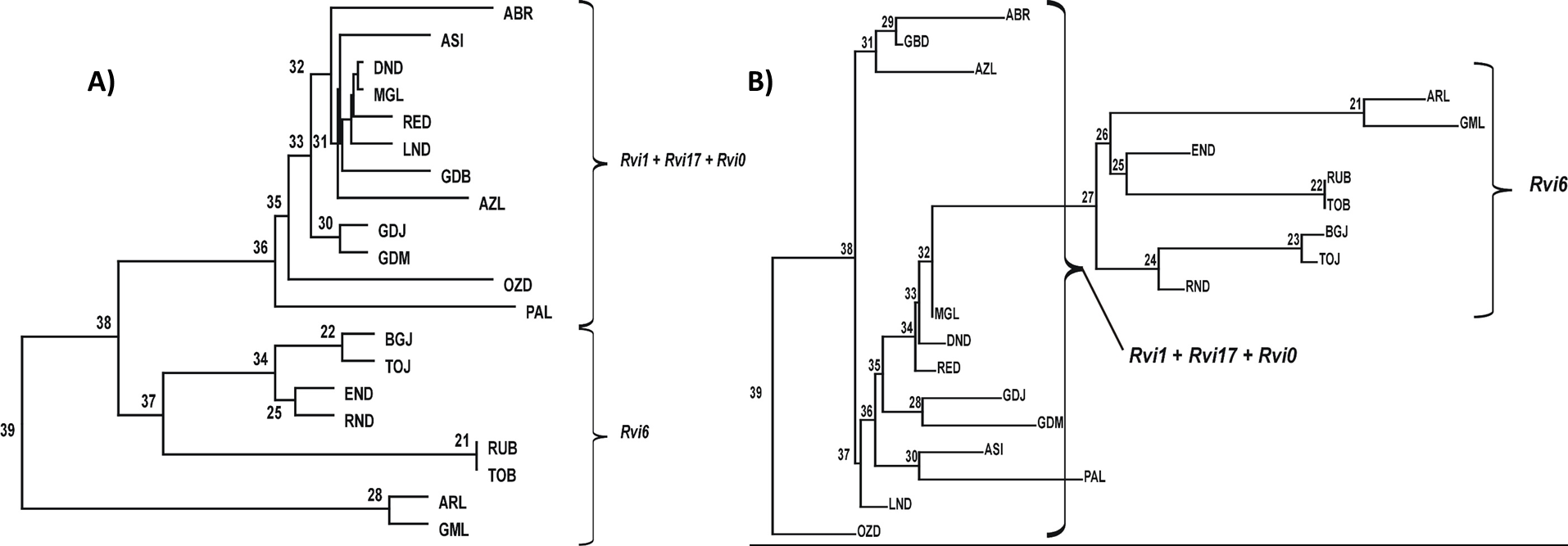

Supplement: Supplementary file 3 — Fig. S3. a) Unweighted pair group method with arithmetic mean (UPGMA) and b) Neighbour-joining(NJ) dendrograms based on Nei’s genetic distance [33] between 20 V. inaequalis populations, genotyped within 11 microsatellite loci. Numbers at major nodes indicate the percentage of times the cluster to the right of the branch occurred among the sample of 1000-bootstrap-generated dendrograms. (TIFF 3745 kb) [file 12862_2018_1122_MOESM3_ESM.tif]
